# “I Don’t Trust it, but I Use it”: Navigating Trust, Privacy, and Identity in Disabled People’s Use of Generative AI

**DOI:** 10.1145/3772318.3790652

**Published:** 2026-04-13

**Authors:** Jazette Johnson, Aaleyah Lewis, Jennifer Mankoff, Olivia Banner

**Affiliations:** Paul G. Allen School of Computer Science & Engineering, University of Washington, Seattle, Washington, USA; Paul G. Allen School of Computer Science and Engineering, University of Washington, Seattle, Washington, USA; Allen School of Computer Science and Engineering, University of Washington, Seattle, Washington, USA; University of Washington, Seattle, Washington, USA

**Keywords:** generative artificial intelligence, GenAI, accessibility, trust, mistrust, privacy, intersectionality

## Abstract

As generative AI (GenAI) is integrated into everyday technologies, it offers new accessibility opportunities and risks for disabled people. However, little is known about how disabled people navigate GenAI in their everyday lives, particularly how trust, privacy, and intersectional identities affect these experiences. We present findings from seven cross-disability focus groups (N=20) that explore how disabled people navigate GenAI. Our findings reveal that while GenAI supports autonomy, efficiency, and communication, it also introduces accessibility taxes and ethical dilemmas. Although participants voiced skepticism, many continued using GenAI out of necessity. Finally, we found identity-based benefits and tensions, in which GenAI preserved and validated intersecting identities, but also misrepresented and erased those identities. We frame these negotiations as a constant balancing act between access and risk, urging research to further examine how “access” is conceptualized. We offer implications for creating GenAI tools that are transparent, trustworthy, and responsive to intersectional identities.

## 1 Introduction

The emergence of generative AI (GenAI) holds potential to support access for disabled people^1^, while also amplifying the risks and biases they face. Several studies have sought to understand how disabled people are using GenAI [1, 5, 9, 13, 30, 43, 65, 68, 86, 95]. While some of this work surfaces intersectional identity markers—such as race, language, or gender—they have done so in narrow, context-specific ways that address at most two axes within single disability groups. Their limitations around small samples, context-specific settings, and the narrow range of disabilities and identities represented limit their generalizability. Additionally, this body of research has not deeply and systematically examined how trust, mistrust, and identity intersect to shape disabled people’s everyday GenAI use.

This gap is particularly concerning given what we know about the complex relationship between identity, trust, and technological adoption. Historically, the disabled community’s trust in technology has been shaped by experiences of exclusion and marginalization in technology development: from assistive technologies designed without disabled users’ input [57] to computing systems that perpetuate ableist assumptions [33, 92]. These historical patterns can create well-founded skepticism about new technologies, particularly technologies that collect data or make decisions about, or for, disabled people. Despite this, many disabled people heavily rely on technological systems for accessibility, communication, and independence—using systems they may not fully trust because alternatives are unavailable. This tension between necessity and mistrust remains underexplored in prior work, despite the high stakes for disabled users, whose autonomy and quality of life are significantly affected by whether these technological systems functioning reliably, without bias or misuse of data and personal information.

For disabled people of multiply marginalized identities, these tensions can be further complicated by additional cultural and contextual context, such as negative historical experiences with technological systems [6, 38]. For example, AI technologies reproduce biases and representational harms—such as misrepresentation, stereotyping, tokenizing, and erasure—across single identity dimensions, including race, gender, language, and disability [2, 21, 28, 31, 37, 47, 50, 54, 78, 79, 87, 93, 96], and these harms have been shown to produce emotional, cognitive, and temporal consequences for users [54]. As argued by Crenshaw and Collins [18, 19], identity dimensions do not operate independently: they interlock and create amplified forms of oppression for multiply marginalized groups. While some research has examined AI’s representational harms through an intersectional lens, disability is rarely included as one of the intersecting axes (e.g., [8, 56, 85]). GenAI’s general-purpose nature, rapid uptake, and integration into everyday technologies allow representational harms to circulate more quickly and widely. Yet examinations of GenAI often treat disability as a single-axis category [28, 31, 54] or at most account for narrow intersections [1, 32, 43], leaving compounding identity configurations unexamined. To summarize, much of this research still reflects a limited subset of tools, disabilities, and use cases, overlooking how factors of trust and identity interplay to shape GenAI use and experiences.

To address the lack of cross-disability research on GenAI’s everyday use and accessibility impacts, we conducted focus groups with 20 diverse disabled participants across diverse identities and experiences to discuss their use or non-use of GenAI tools. Our work answers the following research questions through a qualitative study.

- **RQ1:** How are disabled people using GenAI tools in their daily lives, particularly for accessibility purposes?
- **RQ2:** What factors influence disabled users’ trust or mistrust in GenAI tools?
- **RQ3:** What identity-based challenges and benefits do users with disabilities encounter when using GenAI tools?

We explore the benefits and challenges of GenAI use, conditions that shape trust and mistrust, how necessity and mistrust coexist, and the tensions and harmonies that arise for disabled people with intersectional identities. Our findings provide insight into how disabled people, including those with intersecting identities, use and resist GenAI. In doing so, we contribute to the ongoing dialogue establishing new ways of thinking about accessibility technology as a response to structural inequities [42]. We also highlight identity-specific benefits and risks, extending our understanding of how race, ethnicity, multilingualism, gender, and disability interact in the context of accessibility. Finally, we highlight the additional costs GenAI use imposes on disabled users, deepening our understanding of the concept of the “accessibility tax” [66]. These include financial costs, verification labor, and data disclosure. Our findings will inform the design and study of GenAI tools that reduce accessibility barriers, support diverse identity expression, and foster trust while minimizing the burden on users.

## 2 Related Work

There is growing recognition that accessibility research must meaningfully engage with the breadth and richness of identity rather than apply single-axis accounts of identity. These dimensions do not operate independently but rather are intersectional and can compound to create unique barriers for multiply marginalized groups, as explained by Crenshaw [19]. Moreover, these identities interlock in ways that shape lived experiences and engagement with technology [39]. Harrington et al. [39] proposed a framework for incorporating race into accessibility research; and other workshops have emphasized the importance of grounding accessibility scholarship in disability justice principles [82]. However, this has not yet translated into consistent consideration of intersectional issues in scholarship about GenAI accessibility. In this section, we first show that prior studies on GenAI and accessibility rarely consider how disability interacts with other aspects of identity intersectionally, including race, ethnicity, language, and gender. Next, we consider GenAI use for accessibility. Most early studies of GenAI use among disabled communities were speculative in nature, meaning that they uncover what disabled people want. This work spans neurodivergent communities [16, 36, 45, 63, 84], d/Deaf and Hard-of-Hearing (DHH) individuals [17], blind and visually impaired (BVI) users [26, 44, 89], AAC users [25, 77, 91], people with intellectual or developmental disabilities [74], and disability communities more broadly [23, 32, 54]. However, our focus is on the smaller number of studies that uncover what disabled people are experiencing right now in using existing, deployed, GenAI. We end by highlighting the growing body of work that is beginning to describe what is known about disabled people’s current use of existing commercial tools.

### 2.1 GenAI, Identities, and Their Intersections

Prior research on GenAI has largely taken a single-axis approach, documenting how it reproduces societal inequities and biases along lines of disability [28, 31, 50, 54, 93], race [29, 50, 75, 78, 96], ethnicity [2, 21], gender [21, 47, 78, 90, 96], queerness [87, 90], and language variation [37, 50, 62, 79]. For example, Michel et al. evaluated synthetic AI voice generation services and revealed disparities across five regional, English-language accents, showing how services reinforce linguistic privilege and accent-based digital exclusion [62]. Yet, some single-axis research suggests GenAI can meaningfully support marginalized identities. For example, preliminary work examining ChatBlackGPT suggested the tool’s ability to provide culturally informed responses for Black users [24].

We were only able to identify three studies that address current practices and concerns that relate disability to gender, racial, and/or language diversity [1, 30, 43], all with narrow disability populations. Glazko et al. highlight how identity practices such as code-switching^2^ and masking^3^ were central to neurodivergent users when navigating complex societal norms and stigmas around cultural and linguistic variations [30]. Adnin and Das note tensions in GenAI perpetuating gendered assumptions in visual descriptions and misgendering in outputs [1]. Amidst growing research on GenAI use, there remains limited understanding of how diverse disabled people experience GenAI—particularly in contexts where identity representation is central to user trust and usage.

### 2.2 Current GenAI Use by Disabled People

An emerging literature on GenAI is examining how disabled people are currently using available systems in their everyday lives. This work spans disability groups, with some studies broadly examining disabled communities [5, 9, 65, 68], and others focusing on specific populations, including neurodivergent communities [13, 30], BVI users [1, 86, 95], and DHH individuals [43]. These works suggest disabled people approach GenAI with both caution and enthusiasm—enthusiasm about its possible benefits as an accessibility technology and caution about its risks and potential harms. Several studies have examined everyday GenAI use across disability groups [5, 9, 65, 68]. In early-stage work, Mullen et al. analyzed 18 semi-structured interviews to characterize how disabled people use large language model (LLM)-based chatbots, including ChatGPT, Bard, Gemini, Copilot, and Perplexity, in their daily routines [65]. The authors present a high-level mapping of nine use categories, with writing and ideation being the most common. Communication support is also documented prominently in the studies of DHH [43], neurodivergent [13, 30], and BVI [95] users. Other use cases are more disability specific. For example, household chores, spatial navigation, and fashion selection are important in the BVI community (e.g., [95]) but not mentioned in other studies, while emotional well-being and mental health support are mostly mentioned in studies of neurodivergent populations [13]. Reducing cognitive load and fatigue is a concern mentioned by DHH [43] and neurodivergent [30] users. In addition to characterizing disabled people’s everyday uses of GenAI, these works are beginning to add to our understanding of how identity and trust interact as structuring forces that shape interactions with GenAI. We summarize those themes here.

#### Trust and Risk:

When GenAI is filling an unmet need, studies have found participants make tradeoffs between access and risks—including when they are aware of errors and harms, such as hallucinations during image description for BVI users [1], and negative effects on learning [30]. For example, Adnin and Das [1] found that blind individuals avoided using GAI for high-risk activities such as medical, health, and financial decisions. Tang et al. [86] highlight a skepticism and criticality of blind people toward all forms of information access, including AI-mediated access, all of which require a contextual process of thinking about the politics of access. Yet, there remains a limited understanding of how people with disabilities navigate trust and privacy concerns when they directly conflict with access needs that would otherwise remain unmet.

#### Improvements on Human Alternatives:

At the same time, studies have shown that GenAI is sometimes viewed as better than human alternatives. For example, Glazko et al. [30] found participants trusted that a GAI system, unlike a human interlocutor, would not judge their input, which reduced their anxiety. Similarly, Blind people appreciate the ability to ask multiple questions about images without cost [11]. At the same time, this can lead to a loss of human connection [13], and it risks inhibiting learning and skill development [30].

#### Privacy Concerns:

Stangl et al. [80] investigated the privacy concerns of blind users of visual assistive technologies (VATs) and found greater concern about AI-powered VATs than about human-powered VATs, with significant concerns around how AI-powered VATs handle their data, especially when data is being used unknowingly.

#### Ablesim:

Mack et al. examined disability representation in text-to-image generation models, revealing that available models frequently defaulted to reductive archetypes that reinforce societal misconceptions of disability [54]. In a resume audit study with GPT-4, Glazko et al. compare the model’s ranking of standard resumes against versions enhanced with disability-related achievements (e.g., leadership awards, scholarships). The authors found that GPT-4 systematically ranked the enhanced resumes lower, revealing embedded ableist bias [31].

#### Erasing Identity:

Multiple studies highlight examples of GenAI enforcing norms of communication that elide aspects of participants’ identities, including neurotypical communication [13] and Deaf culture and DHH communication norms [43]. Text-to-image models can also be culturally insensitive (e.g., [29, 74]). For example, Guedes et al. explore the potential of AI-generated artwork for individuals with IDD and identify risks such as misinterpreting cultural elements in generated artwork and cultural insensitivity [74].

#### Accessibility:

In addition to inaccessible interface components reported by screen reader users [95], studies mentioned text-centricity as an access barrier for neurodivergent users [13].

These studies highlight the importance of GenAI among disabled populations and of looking more closely at disability-specific risks, including ableism and how disabled people are negotiating the risks of GenAI use and access needs.

## 3 Study of Everyday, Cross-Disability GenAI Use

To better understand how disabled people use GenAI in daily life for accessibility, how their experiences are shaped by intersectional aspects of identity, and their concerns relating to privacy and trust, we conducted focus groups with 20 disabled people from a variety of backgrounds. While individual interviews are the dominant qualitative method in the field, we chose focus groups as our method because it allows participants to build upon each other’s experiences, potentially revealing insights into GenAI use that might not emerge in individual interviews. Given the historical exclusion of disabled people in the design and development of technology, focus groups have the ability to provide a more empowering research environment, where participants can support and validate each other [48, 49]. Our research questions specifically examine trust, exclusion, and benefits, topics for which both shared community concerns and individual differences are important. We acknowledge that focus groups may constrain participants from disclosing sensitive personal experiences and collected data could be influenced by dominant voices within the group. To mitigate these concerns, we used various moderation techniques (see Section 3.1), created multiple smaller groups to ensure diverse perspectives, and provided opportunities for participants, if interested, to follow up with deeper perspectives.

We were guided by three research questions: *RQ1*: How are disabled people using Generative AI (GenAI) tools in their daily lives, particularly for accessibility purposes? *RQ2*: What factors influence users with disabilities’ trust or mistrust in GenAI tools? *RQ3*: What identity-based challenges and benefits do users with disabilities encounter when using GenAI tools? To address RQ1 and RQ2, we recruited across multiple disability communities (e.g., visual, mobility, cognitive, hearing impairments) and varying levels of GenAI experience, from extensive experience to limited exposure, to understand diverse usage experiences and capture the range of factors influencing trust and mistrust. To address RQ3, we prioritized recruiting participants who could speak to intersectional experiences (e.g., disability + race, disability + language, disability + gender) and included people from varied cultural and demographic backgrounds.

### 3.1 Procedures

We conducted seven semi-structured focus groups on Zoom, each with 2 to 5 participants, for a total of 20 participants. Each session included at least two authors and lasted approximately 90 minutes. Groups were scheduled based on participant availability and included people with diverse disabilities. Two sessions were scheduled specifically to accommodate participants who communicated primarily through AAC devices or text-based chat, with extended time allocated to ensure meaningful participation and in-depth discussion. We began each session with a five-minute introductory presentation distinguishing between the definitions of artificial intelligence (AI)^4^ and GenAI^5^. We shared everyday examples of GenAI, followed by accessibility specific examples. Next, we guided participants through a series of questions that covered daily use of GenAI for accessibility, perspectives on privacy, safety, and trust when using GenAI tools, and perspectives on how GenAI has, or has not, represented their intersecting identities (Appendix A). After data collection was finished, we asked participants to complete an optional survey to collect additional background information on their occupational, educational, and technical backgrounds (Table 3). Ten participants completed the survey.

After receiving IRB approval from our university’s ethics board, we recruited participants through U.S.-based community organizations serving disabled people and snowball sampling. Initially, we contacted seven community organizations; we ultimately received participants from four organizations, including Diverseability, People’s Hub, National Federation for the Blind, and Divas with Disabilities. People who were interested in participating in the study completed a Qualtrics screener survey that asked questions regarding basic demographics (e.g., race/ethnicity, age, language, and disability identity), and experience level with/knowledge of GenAI. To be eligible to participate in the study, individuals had to be 18 years of age or older and self-identify as having a disability or chronic illness. GenAI use was not a requirement because even people who choose not to use GenAI encounter it in a variety of settings. Each participant received a digital Amazon gift card, its monetary value reflecting session length (60 minutes = $35; 90 minutes = $50).

Recruitment occurred in multiple phases, prioritizing disability and multicultural backgrounds. Some phases were significantly impacted by scam screener responses, a common challenge in online recruitment [34]. We identified scam responses through suspicious patterns (e.g., emails sent under a different participant’s identity) and implemented strengthening measures, including manual review of responses, requiring that cameras were on during interview sessions, and targeted organizational outreach. If scammers got through to complete interviews, the full group data were removed to eliminate possible scammer influence on other participants.

### 3.2 Participant Demographics

Participants in our study represented a diverse range of disabilities, including blind or visually impaired (N = 6), chronic illness (N = 8), d/Deaf or hard-of-hearing (N = 5), intellectual or developmental disability (N = 2), mental health disability (N = 4), motor disability (N = 3), neurodiversity (N = 8), speech disability (N = 2), AAC user (N = 1), and multiply disabled (N = 10). Participants ranged in age from 18 to 85 years. Most participants had prior GenAI experience, with only three exceptions.

The remaining participants (N=17) reported using GenAI tools, including chatbots (e.g., ChatGPT, Perplexity, Claude), image generators (e.g., DALL-E, CanvaAI), and image description/object recognition tools (e.g., BeMyAI). Additional demographic information is presented in Table 2 and Table 3.

### 3.3 Data Analysis

We used a mixed-method approach to analyze our data, using descriptive statistics and reflexive thematic analysis [12]. We conducted descriptive statistical and thematic analysis of participant demographic data collected through the screener survey and the follow-up demographic survey. This included calculating frequencies and percentages for categorical variables (e.g., race/ethnicity, disability identity, prior GenAI experience). Before calculating frequencies, we conducted deductive analysis on self-reported disability identity to group them into higher-level disability identities. We also conducted an inductive analysis on self-reported GenAI use. Participants used GenAI and AI interchangeably when discussing GenAI, but we only included examples of GenAI in our final analysis.

We conducted reflexive thematic analysis [12] of focus group data using an iterative, multi-phase approach. Two researchers began with inductive coding, being cognizant of emerging patterns in the data. They independently reviewed focus group transcripts, generating initial codes that captured semantic and latent meaning in participants’ discussions about using GenAI. Next, using affinity diagramming, the researchers grouped and discussed codes to create higher-level groups for the codebook. Finally, the researchers used a mixture of deductive and inductive analysis, using the codebook to identify themes while also remaining open to new codes and themes that emerged. After final grouping and team discussions, we iterated a final time on the codes. Throughout our analysis, we prioritized themes that were both prevalent across participants and related to our research questions. Throughout data collection and analysis, we used our research questions as touchstones for methodological decisions, ensuring that our approach remained focused while still allowing for the discovery of unexpected insights.

### 3.4 Accessibility Considerations

We anticipated that participants would have diverse access needs. To support this, we began each focus group by establishing accessibility norms, which included saying one’s name before speaking, taking breaks as needed, muting when not speaking, and enabling captions for all sessions. Participants were also asked about access needs during the screener survey, while scheduling their focus group session, and again at the start of each session. In response to these requests, we provided accommodations such as interpreters, sharing each question in the chat, allowing participants to respond to questions in the chat, and distributing interview questions in advance.

### 3.5 Positionality

Our authors are all based in the United States and include disabled people, women, queer folks, and those who variously identify as White, Black, and Jewish. We acknowledge that our understandings of disability, accessibility, race, gender, and sexuality are informed by our positionality and experiences. Moreover, our recognition that accessibility is shaped by intersecting systems of power guided our analysis. Our team holds varied relationships to GenAI: three authors actively use GenAI—ranging from daily to monthly use across accessibility, professional, and personal contexts—while one author actively resists using it. These differing experiences with GenAI informed our understanding and analysis of the risks and benefits surrounding GenAI use. Specifically, authors with GenAI experience brought insider knowledge of tool capabilities and limitations, while the non-user questioned assumptions about GenAI benefits and ensured equal attention was given to participant concerns about risks, privacy, and exclusion.

## 4 Findings

Our analysis of seven cross-disability focus groups revealed that participants’ experiences with GenAI were shaped by a constant balancing act between accessibility gains and structural risks. While we found many of the same uses for GenAI described in prior work, our findings also highlight new concerns. Participants emphasized the tradeoffs they navigated, including: *maintaining autonomy by compromising safety; negotiating GenAI outputs across identities*; and *leveraging efficiency gains while paying accessibility taxes*. Throughout our conversations, participants highlighted both the empowering potential of GenAI and the new accessibility taxes, uncertainties, and ethical dilemmas it introduced. They also described *strategies for managing GenAI costs*.

### 4.1 Current GenAI Use and Affordances

Participants used integrated GenAI tools (e.g., BeMyAI, CanvaAI, Google AI overview) and standalone GenAI tools (e.g., ChatGPT, Claude, Dall-E). Consistent with prior work [32], use occurred across four primary domains: as a creative outlet, for professional and academic support, for information seeking, and for accessibility assistance. Notably, accessibility assistance was often present among the other three domains.

Also echoing prior work ([30, 43]), GenAI tools were frequently used to navigate normative communication expectations, simplify text, and generate text. Participant satisfaction with GenAI was closely tied to its ability to affirm identity and linguistic preferences. For example, Alexander explained how the tools *“generalized”* his communication style: *“I don’t type correctly. I don’t use full sentences, I just use abbreviations. Sometimes curse words. And I’ve noticed that irrespective of however I shape my prompts, my question, I get [a response] in a refined format that’s like in a standard format… I just get a generalized answer… ”* Alexander praised the tool for consistently producing generalized outputs that uniquely supported his communication style by not judging how his inputs were written.

GenAI was also described as efficiently supporting tasks. Thomas explained that, compared to SeeingAI, BeMyAI improved his experience: *“Once I started using BeMyAI like that, I just felt so much less stress. I didn’t have to take the picture over and over again without knowing what the problem was because … it told me like ‘Your thumb is covering the camera’. And I’m like, oh, I didn’t know that.”* GenAI reduced Thomas’ stress by providing clearer feedback and streamlining his access task. This appeared in various settings. For example, Nicole expressed how GenAI tools addressed professional barriers that had persisted for years: *“As long as I give it the detailed instructions that it needs, it can generate visual and coded prototypes. And as a blind designer, man, that’s been an obstacle for years.”* Amy created her own solutions when her school failed to provide support: *“I use [AI] on Zoom for transcription purposes because the school did not provide that. So it was helpful for me because I might have had to retake that course.”* Many accessibility tasks, even when possible, are inefficient because they require extensive effort or repeated trials to be completed. GenAI is filling a gap in available accommodations, enhancing participants’ capabilities and addressing institutional and structural failure.

### 4.2 Navigating Mistrust: Using GenAI Despite Systemic Risks

When participants used GenAI for independent decision-making, it increased their autonomy by helping them to complete tasks independently and supporting self-determination. However, this benefit required compromise. For example, Kim explained: *“I literally trust GenAI tools because I use it literally every day. It’s like another replica of me because I need it to read, I need it to literally even talk to someone, I need it to do a lot of things*.” This shows the necessity of use rather than genuine confidence in a system’s reliability or privacy protection. No participants trusted GenAI unconditionally, but instead participants were cautious users of GenAI technologies.

While many participants embraced the use of GenAI, they often expressed reservations about GenAI’s trustworthiness. Beyond technical and safety concerns, participants expressed concerns about broader systemic issues such as privacy, environmental impact, and corporate ethics. Participants described using consequence calculus^7^ [52], navigating tensions between their accessibility needs, safety, privacy, and their ethical concerns. When GenAI is necessary for access or safety, this exposes disabled people to increased risks they otherwise would not take. Participants held a range of complex positions, balancing their accessibility needs, personal privacy, and safety with their reservations about AI, with attitudes ranging from *conditional trust* to *active resistance*.

#### Reliability Concerns:

GenAI errors and bias put participants at risk of serious indirect consequences, particularly in situations that require precision or when users have limited capacity for verification. Thomas, who is blind, described his concerns about using GenAI in financial activities: *“So when I was scanning [money]… I remember I used to have to line up the bill just right so I could get it right, but I was scared that what if it says it’s a 20, but it’s actually a 200 … ”* Lex elaborated on how accuracy concerns intersect with accessibility needs: *“Think on an accessibility standpoint, that could be kind of dangerous depending on what you’re asking the AI for and there could be problems that way accessibility-wise.”*

Despite these reliability concerns, some participants developed conditional trust strategies — using GenAI under only when the risk is “very low stakes,” as Michelle notes: *“I think, overall, I tend to trust GenAI … more when it’s a very low stakes outcome. For instance, if it’s something that I know I’m going to tweak in the end, I’ll take it for what it is, but still make my adaptations where it’s necessary.”* Other participants described only trusting GenAI once they can verify its reliability through ongoing conversation: *“Where I do trust it, is when I can start to ask deeper questions and really get specific about what I’m wanting to know. And once I’ve dug a little deeper into the conversation and I feel like it’s locking in, we’re good to go and I’m on board.”* These participants actively construct trust through either limiting exposure to risk or conducting their own informal reliability test. Each of these strategies places additional cognitive and temporal burdens on users who may already face barriers in accessing information through traditional means, which we explore more in Section 4.4.

#### Environmental Harm and other Ethical Concerns:

Participants expressed concerns about the environmental costs of using GenAI tools, viewing their excess energy and water consumption as an ethical issue that created internal conflicts when GenAI was necessary for access. Lex described these tensions directly: *“There’s also a lot of environmental and ethical standpoints on how these AI bots are trained. That makes me very wary of them [although] I do need them at this point.”* The phrase “I do need them” indicates the use of consequence calculus—environmental concerns could not override immediate accessibility requirements in a world offering few alternatives.

For some participants, ethical concerns were significant enough to lead to active resistance. Sophia described her rejection of GenAI as partly motivated by these concerns: *“I don’t like how it uses so much energy and water. … ”* However, Sophia’s ability to resist suggests access to alternative means of access that not all participants possessed.

#### Corporate Ethics and Mistreatment of Disabled Users:

Participants also expressed mistrust in corporate ethics, sometimes informed by previous negative experiences with proprietary access technologies. Ethan explained: *“Sometimes it is about the business behind it for me… from a privacy standpoint, but also accessibility and how they treat/handle people with disabilities… ”* Similarly, Sara echoed concerns about corporate practices: “I have not seen an active involvement of including the disability community in these AI tools and knowing the politics.” Sophia explained, “… we don’t know the motives or we can’t suspect motives of the people who control it, and we’re really kept in ignorance and they aren’t really open to the public. I’m not really happy with it.” For participants with intersecting marginalized identities, mistrust was compounded by historical harms done to their communities. Fran’s concerns centered on LGBTQ safety and organizational competence: *“I do have hesitancy around using certain types of GenAI tools because of opinions that leadership of different companies have… I’m an LGBTQ person, so the ways that people talk about LGBTQ people, I do get a little worried about using some generative AIs from companies that are not competent.”*

Historical harms done to specific communities may impact trust differently for those groups, compounding to discourage use even from those who might safely benefit. For example, Lex, an Indigenous and Mexican participant, described encountering content they characterized as “super racist,” and situated their mistrust in historical patterns of technology enabling the oppression of marginalized communities, reinforcing fears that GenAI could further marginalize their communities. She has, *“… a lot of hesitancy when it comes to new technology, mostly because a lot of new technology has been used in ways that hurt a lot of my community… With all of the access to information that they have, they can find out ways to hurt us better.”*

Yet, despite these profound concerns about corporate accountability and historical patterns of technological harm, participants continued using GenAI because it provided access unavailable through other means. Fran described the importance of GenAI in the face of access barriers: *“I think generally I don’t trust AI, but it’s kind of a means to an end… [it] creates access in a world that is fundamentally inaccessible … I ultimately still use it because it is informing my life in ways that I’ve not had with other types of accessibility tools.”* This continued use despite mistrust reflects not trust-building, but the absence of alternatives in systemically inaccessible environments.

#### Privacy and Data Security: Uncertainty and Continued Use:

Uncertainty about technical decisions, such as model training practices and privacy safeguards, also created mistrust. Lex expressed: *“Some of the AI stuff that I’ve seen, just like horror stories kind of freaks me out. But using it … cause I don’t know if it’s been trained ethically, that’s the problem.”* GenAI mistrust was also expressed due to the lack of safeguards protecting user data and preventing misuse. Jasmine described: *“It feels like we can’t trust them. I think I might trust them a little bit more if they had stricter rules and safeguards and they could ensure that our information was a hundred percent protected, that they could spoof our voices, they couldn’t steal those types of things from us. But for now, I don’t use it for that reason. It doesn’t feel a hundred percent safe.”* Amy worried that data is *“probably being sold or being used for other things, probably being sold to companies or things like that. So I do not trust any GenAI tool actually.”*

However, many participants who expressed these privacy concerns continued using GenAI when accessibility needs or real-world consequences outweighed their reservations. Emory described the tradeoff between sharing personal data with GenAI and real world consequences such as finding work: *“I actually really don’t trust [GenAI tools] as kind of the baseline, which doesn’t mean that I don’t use it … If I didn’t have such a hard time finding work and feeling like I could do all of the performance code-switchy, … but I do need to.”* Platform selection sometimes reflected attempts to manage privacy risk within the constraints of continued use. Maddy explained how she selected which platforms to trust: *“When I look stuff up on Google, I trust [GenAI results] because it’s Google, but when it’s like the MetaAI, I’m not trusting that one yet because it’s a newer type of AI.”* Participants worked to mitigate GenAI problems when their immediate accessibility needs outweighed their reservations, but understood the limitations of the mitigation option available to them, creating tensions where mistrust coexisted with continued use.

#### GenAI as Necessary Despite Mistrust:

For many participants, GenAI was not merely convenient but necessary for access, safety, and social legitimacy in normative environments — making mistrust a burden they had to carry rather than a reason to discontinue use. Ethan valued GenAI’s detachment from the emotional complexities that often accompanied human assistance: *“… I have to train a person a lot just to know how to be with me sometimes because they can get nervous or upset about things they don’t understand too. Conversely, I never have to tell AI ‘ignore this crazy looking device I’m using’ because the AI is not something I’m developing a relationship with that is emotionally reacting and involved.”* GenAI can provide judgment-free support, reducing the social and emotional labor often involved in human support. For Lex, GenAI became essential for emotional safety in professional communication: *“I have a hard time writing professional emails … I had one professor yell at me and say that I was being super unprofessional in the email … So I’ve started writing what I would normally write and then porting it in to ChatGPT and say, ‘Make this a professional email,’ so I don’t have to get in trouble by the people that I am talking to ‘cause the way I say stuff isn’t good. So it helped me a lot. I had that one professor or two of them really pop off on me for lack of professionality in my emails.” Here, GenAI use wasn’t driven by trust in the technology, but by fear of social consequences for neurodivergent communication styles — a need created by systemic ableism rather than genuine technological preference*.

#### Active Resistance:

We define resistance as the active refusal to engage with GenAI tools. While some participants described varied relationships with GenAI, such as forms of selective or situational engagement, we do not conceptualize these practices of negotiation in technology adoption as resistance. Rather, we situate resistance around the explicit non-use and rejection of GenAI technologies. Only three participants did not use GenAI. As described above, Sophia’s resistance centered on environmental and transparency concerns, while Bond expressed fear of GenAI misusing personal information: *“I am afraid that AI will somehow use its wits to jeopardize our personal information. I hope this is not the case, but I’m afraid that this is something that might happen in the future and that’s something we need to take seriously … ”* These perspectives represent a deliberate resistance to GenAI, highlighting how concerns about corporate accountability, environmental impact, and data privacy can outweigh utility.

### 4.3 Negotiating GenAI Outputs Across Identities

Participant identities (disability, race, ethnicity, language, gender, and their intersections) shaped how they used and experienced GenAI tools. Participants reported interactions where GenAI tools preserved and/or validated their identities and their intersections, while others reported that tools failed to honor their intersecting marginalized identities, requiring participants to employ workarounds or avoid use altogether and increasing their mistrust of GenAI tools. Prior work has documented how GenAI erases or stereotypes marginalized identities [50, 54]. Our findings extend this literature by distinguishing between *identity preservation*, *identity validation*, and *supportive code-switching*.

#### 4.3.1 Preservation of Identities and Their Intersections.

Ideally, accessibility technology would preserve participants’ unique identities without erasure or collapsing into dominant norms (i.e, White, male, Standard American English defaults). Identity preservation successes and failures arose in domains ranging from multilingual support to recognition of disability, racial, and/or gender identities. When GenAI fails to preserve identities and their intersections, it risks structurally reinforcing inequities and undermining holistic access. Some participants had positive experiences with identity preservation, but participants also reported problems with identity preservation due to omission or normative assumptions. As a result, accessibility requires compromising other critical aspects of identity, or additional accessibility taxes in cases where problems can be overcome.

##### Multilingual Support:

Multilingual and multidialectal support includes the preservation of linguistic variation in inputs and/or outputs. Thomas, a bilingual English-Spanish speaker, described ChatGPT bridging both his language and visual access needs by generating translations in both directions: *“I’ll scan things that are in Spanish and [ChatGPT] will read to me in Spanish. And so I’ll ask if [ChatGPT] can translate it.”* He also described using ChatGPT to support his Spanish-speaking mother, highlighting interdependence [7]: *“I’ll do the same thing the other way around… my mom needs help with something, and then I’ll just scan it and … read out the text through English, and then I’ll ask [ChatGPT] if you can translate it for me and then it’ll send it that way.”* Here, GenAI enabled Thomas to meet his own intersectional access needs and to extend that access outward to his mother, facilitating reciprocal care. ChatGPT also provided Thomas with image descriptions in Spanish: *“I can dictate it or type it into the ChatGPT any question about an image, [ChatGPT will] describe the image in Spanish, which is always nice.”* Amy recalled GenAI supporting low-resourced language output, *“I saw people doing a trend of [GenAI] responding in Pidgin English. So I tried it and it worked … the fact that AI can respond in Pidgin English for people who actually want it to do so is something good.”* Together, these underscore the importance of preserving diverse linguistic identities in GenAI interactions—not only to reduce barriers to access but also to allow participants to achieve access while maintaining their authentic way of speaking, writing, and presenting themselves.

##### Erasure of Linguistic Identity:

For participants whose linguistic practices are low-resourced in computing, GenAI tools often failed to honor their linguistic practices, thereby reinforcing societal language hierarchies [73]. For example, Jasmine, a multilingual user who primarily communicates in ASL, would like to sign in ASL and receive English output from GenAI, but avoids using it because current tools are speech- and text-centric: *“It’s awkward … [GenAI] doesn’t understand sign language. So for me, it’s not as intuitive as for most.”* She characterized many GenAI tools as not being Deaf-friendly, lacking awareness of Deaf culture, and marginalizing her primary language of ASL by privileging English, which for her is a second language. Similarly, Ann reported that *“text-to-speech with my accent, it’s not always good.”* This highlights the intersectional nature of accessibility taxes. Ann uses text-to-speech to alleviate chronic pain from prolonged typing. While this provides a good starting point to get her thoughts down and helps speed up writing, she is forced to do extra work because of her linguistic variations.

##### Normative Defaults:

Consistent with prior work, participants with intersecting marginalized racial identities reported that GenAI systems reproduced racialized bias. When identity was unspecified, outputs defaulted to monolithic portrayals, normalizing Whiteness, a structural bias that collapses identities into singular, dominant portrayals [69, 88]. For example, when using GenAI to help generate images for social media posts, Sara shared, *“When I don’t specifically say what the gender is, what the ethnicity [is], and I just type something it does bother me when it’s like a White male most of the time… I haven’t really found anything that I would consider multicultural, multi-identity default.”* The absence of identity specification triggered outputs that reproduced dominant cultural norms. Tonya echoed this—during information seeking tasks such as inquiries about her diabetes, when she didn’t specify her identity as a Black disabled person she received outputs that appeared to present themselves as “neutral,” yet when answering critical questions, *“it does not really go down to a Black person… that does not relate to me.”* This supposed neutrality is not neutral at all—it encoded dominant assumptions by universalizing Whiteness and treating the baseline user as implicitly White.

#### 4.3.2 Validation and Affirmation of Identities and Their Intersections.

Validation of identity occurred when identities were reflected back, understood, seen, or valued once shared. However, distorted, stereotyped, or reductive portrayals compromise access and strip away the intersectional nuances of disabled lives.

##### Disability Identity Affirmation and Resonance:

Participants highlighted moments where GenAI recognized and affirmed their disability identities. For example, Tonya described experiencing GenAI honor her disabled identities through personalized, identity-aware responses. She shared, *“I actually believe that AI or generative tools have actually been honoring my identity as someone with a disability. … I’ve actually asked questions in regards to my disability and then telling it that I’m a person with a disability, and then it’s always ensuring as if it’s talking to someone that has my kind of disability.”* For Lex, GenAI provided a rare feeling of being truly understood, particularly for communication styles that are tied to their neurodivergent identity—which prompts their use for interpersonal communication support: *“How I speak is not something that people can understand when I’m just talking like that. And I’ve been able to just voice-to-text into ChatGPT and it understand what the fuck I’m saying and respond back to me with hardly any, ‘What do you mean by that?’ or, ‘That did not compute’.”* She also shared that when using it to help her write professional emails, the tool’s ability to understand her communication patterns *“when other people have issues is a little validating.”* These positive experiences were amplified when GenAI validated multiple aspects of participant identities. Kim, a multilingual Spanish-English speaker, praised ChatGPT’s ability to sustain multilingual interactions, which provided a sense of companionship: *“Having a genAI who speaks Spanish back to you, it’s like, wow. I just feel like I have my own person, like I have someone I can just talk to.”* She emphasized, while human assistance is deeply appreciated, it makes her “feel more disabled,” highlighting the emotional and social costs of human help [20, 35]. GenAI alleviated relational stigma and burdens while it affirmed disability and linguistic identity and offered emotional resonance. GenAI’s ability to understand participants without requiring them to modify how they communicate was transformative because it made them feel seen and understood on their own terms.

##### Stereotyped Racial Representations and Racialized Language Assumptions

GenAI represented diversity superficially and reductively. For example, Ann, who has chronic pain and uses GenAI to assist her in making creative content, recalled an instance using ChatGPT: *“I was trying to create an AI graphic, like a Christmas graphic of black women doing ornaments, but it outputted the same type of black women and it didn’t sit right with me.”* Similarly, when using ChatGPT to create captions for an annual youth conference she leads, Michelle encountered stereotype-congruent language, *“It used a lot of slang terms that would be offensive if a Black youth were reading this.”* In both cases, the tools reflected identity back to participants, but in ways that misrepresented and distorted it. Such single-archetype and stereotyped representations erase intra-group cultural variation. Race, ethnicity, and language biases are intricately linked and mutually reinforcing [3, 4]. As a result, GenAI systems embedded racialized assumptions into linguistic processing, positioning language itself as a site of epistemic exclusion and injustice [22, 27]. For example, Thomas noted stronger and consistent results for technical queries when asking in Japanese and English *“what a string of Python will do”* versus when asking in Spanish, which instead gave him *“information back about pythons as in the animal.”* He attributed these different responses to these systems’ embedded racial and ethnic biases and to inequities in who builds GenAI systems.

##### Gendered Misrepresentations and Normative Conflations:

GenAI also reproduced normative and monocultural gender assumptions. For example, Sara noted that when using it to support writing and creative content tasks, the system altered LGBTQ+ terminology: *“It sometimes will flip ‘non-binary’ with the word ‘queer’.”* This conflation of terms for distinct identities reflects heteronormative assumptions and cisnormative reductions, erasing critical nuances in meaning and undermining self-representation. Sara also noted difficulties with sexist output when she used GenAI for writing and creative content task support: *“I do struggle even with blog posts and making sure that if I say I’m a female, making sure it’s not sexist. And of course the lack of multiculturalism when it comes to that.”* GenAI did not simply overlook her identity: it distorted it in sexist or monocultural ways.

##### Supportive Adaptations:

In some cases, participants viewed GenAI-initiated communication norming as a supportive form of code-switching rather than as identity erasure. For example, some participants felt supported when GenAI outputs had a “neutral” or standardized tone when their inputs reflected neurodiverse communication styles, as noted in section 4.2. For some, these interactions intersected deeply with their linguistic identities as well. For Alexander, who identifies as dyslexic, African American, and who writes as he speaks, *“with people around me who are in my community”*—using slang, abbreviations, and sentence fragments—GenAI-mediated code-switching felt affirming. Even when his inputs included linguistic variations, the system still reliably understood his communication patterns. Alex did not need the system to respond using his communication patterns; instead, it outputted Standard American English, which he saw as a benefit. Rather than forcing him to manually code-switch, the system met him on his own terms and adapted on his behalf, providing “refined” outputs without penalizing him for linguistic differences.

Together, these findings reveal the non-uniform nature of positive identity support, which may include preservation, validation, and distortion of identity through support for code-switching and masking. Importantly, each manifestation carried distinct intent, with participants not only seeking communication support but also affirmation, companionship, and social legitimacy. However, erasure, stereotyping and other types of distortions added to the burden of GenAI use and identity work that participants faced.

### 4.4 Leveraging Efficiency Gains while Paying Accessibility Taxes

The concept of a “crip tax” was first mentioned in academic settings in 1999 [94], though it existed earlier in a non-academic context^8^. More recently, Olsen et al. [66] separated what they term “disability tax” from “accessibility tax,” defined as a form of cultural taxation “unique to disabled people who face inherent barriers to simply using the technologies of physical and virtual work and must pay the direct and indirect financial costs as a result of inaccessibility.” While Olsen et al. connect accessibility tax to technology, the concept is underexplored in accessibility literature^9^, although it is conceptually closely related to the notion of consequence calculus [52]. Additionally, these concepts have not yet been connected to GenAI use.

In our findings, participants continually navigated challenges with GenAI use through employing various adaptive strategies, which incurred cognitive, temporal, and financial costs. These forced costs represent an *accessibility tax* that participants must pay to meet their own access needs, or to receive the same benefits from GenAI as others receive without the same cost. These included financial costs, disclosure costs, and additional labor, such as prompt engineering, to improve the quality of GenAI output.

#### Financial Accessibility Taxes:

Current GenAI pricing models create tiered accessibility where financial resources determine access quality. Amy described the difficult choice between essential features and financial sustainability: *“When you’re using ChatGPT and you’re not on the pro version… there’s a limit on things you can do within a period of time … that is very limiting. [I’m] currently not getting paid for work so it’s a steep slope for me paying for pro.”* Morgan’s calculus to weigh financial costs when deciding whether to use VOCR10 to enhance vision accessibility illustrates this complexity: *“I actually find that that starts to head down a slippery slope of financial craziness … When you have to start buying tokens and points and there’s no real knowing how much a query or a prompt is going to cost. That’s a problem to me. So that’s where I lean away. So if I can’t do it for free or within my little subscription package, that’s as far as I go.”* Sophia described the time, cognitive, and financial costs of a transcription tool: *“Sometimes [Otter] doesn’t connect and sometimes it transcribes not very good, it’s not understandable, or not quick enough. And then you have to pay if you go over a month or so. So that kind of stops me. If they’re not dependable, not accurate, doesn’t help me, I’m not going to pay for it.”* These experiences demonstrate how current GenAI pricing models can inherently create tiered accessibility, where financial resources determine the quality of access support available to disabled users.

#### Disclosure of Personal Information:

For many participants, using GenAI required sharing personal information. Some participants were willing to disclose personal information and disability status as long as it did not include highly sensitive data such as Social Security Numbers, addresses, or income details, while others reported using pseudonyms, avoiding uploads of important documents, or limiting queries to “low-stakes” tasks. These behaviors reflect a lack of trust in data handling practices. Most participants were comfortable sharing general personal details while remaining protective of their sensitive information. For example, Ann was concerned about lasting privacy implications if certain information was used as training data: *“So personal data where it’s not personal-personal data, like social data or where you live at, your income status, all those things. Not like that [won’t share that information], because that training can be harmful.”* For some participants, such as Thomas, privacy concerns created barriers that limited their use of certain GenAI tools and features: *“That’s been the biggest barrier is confidence in the whole idea of being able to protect your private information. Personally, I haven’t really used a lot of this type of AI for that purpose just because of that, just because I don’t know what’s on the document, and so I might just have somebody read it that I trust.”* To protect her privacy, Maddy used alternative names: *“Well, some AI you can’t really trust yet, I don’t really reveal that personal data when I use it. I use different name when I use it so I don’t reveal my data or info.”* These findings reveal how participants paid for access through workarounds or sharing data they preferred to keep private.

#### Disclosure of Disability:

Many participants were far more willing to disclose their disability to GenAI tools than other personal information because it was necessary for high-quality GenAI results. Kim expressed this view: *“I don’t think [GenAI] can help me if I can’t share my disability with [GenAI tools], so I don’t have any concerns because I really need help.”* However, most participants were not strongly concerned about disclosure, even though disclosure is a sensitive topic for many people with invisible disabilities [55, 64, 67, 83]. Tonya explained: *“I do not have any concerns with sharing my disability with generative AI tools, and I share my disability because that defines me, and generative AI tools could actually help. I also have ADD … so generative AI could also help in answering questions I actually generally [need] answers to… I do tell generative AI or ChatGPT that this is who I actually am, that I have ADHD, so it could know how to answer my questions to my understanding. I don’t have any concerns.”* Many other participants similarly viewed disability disclosure as essential for effective GenAI support, outweighing privacy concerns.

#### Prompt Engineering:

Participants described being specific and modifying prompts repeatedly, with Lex explaining:*“With all of my emails … I’ve started just writing what I would normally write and then porting it into ChatGPT and say, ‘Make this a professional email.”‘* However, participants feared the output would lose their authentic voice, raising self-erasure concerns. Nicole described how repetition was often necessary with GenAI: *“Telling AI that I’m blind, I have to remind it sometimes because I need it to give me information that I need. I had a form that I somehow didn’t know what was selected and what wasn’t. I took a screenshot and I said, ‘What is selected?’ And it goes, ‘The items with the red check marks next to them are selected.’ I said, ‘Hey, genius, you’re talking to a blind person’.”* Despite iterative prompting, some participants found that GenAI overlooked identity details, resorting to monolithic defaults. Sara described trying to diversify the racial and gender identities of characters in generated images through re-prompting, but *“it’ll just repeat the same white male”*. Ann reported that avoiding stereotyped results required exhaustive specifications and found it troubling that diversity only emerged when she over-described identity. She noted it was *“a little weird how I would need to be descriptive for [the GenAI model] to not output the kind… that it gave.”* Michelle also commented on the amount of effort required: *“I had to keep giving it prompts to rephrase it and readapt it. I’m like, ‘I shouldn’t have to put in this much work.’ I could have just, what I came up with myself, I could have just kept that.”*

#### Priming and Training:

Multiple participants worked to engineer solutions that could last across prompts. For example, Ethan mitigated misrepresentation through tool choice and personalization, adopting locally trained models over publicly-used alternatives: *“My AI, I train myself locally. I don’t have a larger public dataset bias in interpreting what I ask.”* He acknowledged that such systems *“develop [their] own bias, just not a systemic one,”* trading broad corpus biases for a profile tailored to his experiences. Tonya detailed a deliberate priming process to elicit more competent and representative responses to counter White-centered replies. She shared, *“If I’m asking questions in regards to myself, I tend to be very specific, I define myself. I ask a question, ‘As a Black person,’ since I’m actually Black and a person with a disability. I make sure that I actually narrow the question down to myself and then I explain to AI.”* She explicitly discloses *“I’m a Black person with a disability so it could actually know how to answer my question effectively,”* a strategy combining prompt engineering with disclosure.

#### Quality Engineering within the GenAI Ecosystem:

In cases where participants could easily check and improve GenAI outputs themselves, they did. Fran described a strategy of using different AIs to improve on an output: *“I’ll start with ChatGPT and then give that answer to Claude … I feel like Claude is better at writing personable writing just generally, but ChatGPT does a better job at summarizing information.”* However, the need for cross-referencing creates additional barriers that may not always be feasible. As Lex noted, *“A lot of people don’t do the extra step to fact-check … and if you’re in a situation where you have limited spoons … some people don’t have an option, they just need to do something really quick.”* This highlights how quality engineering can become an accessibility barrier [32] that forces users to choose between speed and accuracy.

#### Explicit and Implicit Human Support for Verification:

When a source document or GenAI output is not accessible, errors add to the accessibility tax [32]. Participants described verifying AI responses by asking family/friends, sighted helpers, or healthcare professionals, or using other AI tools or Google searches. Tonya explained: *“At times, I take the questions to Google to confirm [accuracy]. I mean, I compare and contrast to know if I would actually get the same answer, and that helps in determining if the information from ChatGPT is very accurate or at times, is actually wrong.”* Participants also described asking sighted helpers, family, or friends, with Thomas noting: *“It was a lot of work in a sense to make sure it was all right … I’ll run that by a real person … there’s something about being able to have certainty with somebody who’s there and has sight.”* For health- and disability-related information, participants mentioned healthcare professionals as the verification source, as Kim described: *“So since I’m using genAI,… most times I do the kind of the compare and contrast. If I try to get information from my GenAI and when I get to meet with my GP (general practitioner, like a doctor), I tend to ask almost the same question to see if I’m going to get similar things.”* This type of implicit support allows Kim to avoid the potential costs of disclosing GenAI use and to minimize the perceived cost of support by integrating it into a conversation that would have happened in any case. Yet, this still incurs cognitive and temporal costs, as it requires triangulating information across systems—adding mental load, delaying confirmation, and consumes limited consultation time.

## 5 Discussion

Our findings illustrate how disabled people’s everyday interactions with GenAI are marked by a constant negotiation between opportunity and constraint. Participants embraced GenAI for the efficiency and autonomy offered, often in the face of ableist individual behaviors and institutional failures. However, these benefits required constant compromises, including using untrustworthy technology, overlooking environmental and corporate harms, and lack of privacy and data security. In addition, participants described negotiating identity preservation and validation. To manage these tensions, participants paid a variety of accessibility taxes, ranging from financial cost to additional labor in prompt engineering, quality engineering, and leaning on friends and family for output verification. As a result, GenAI use was not a fixed decision, but a process of weighing benefits against risks and ongoing boundary-setting.

Taken together, these insights highlight how access through GenAI is never straightforward but is entangled with mistrust, risk, and identity-based harms. In this discussion, we situate these findings within broader HCI scholarship and outline implications for designing GenAI systems that include *designing for mistrust, accessibility in and through identity management, and reducing accessibility taxes*.

### 5.1 Designing for Mistrust

Disabled people rely on GenAI, despite doubts about accuracy, fairness, or privacy, because accessibility benefits often outweigh risks. The dilemma between mistrust and continued use shows how disabled participants are often forced to navigate potentially risky technology, not by choice, but by necessity. This necessity reflects systemic barriers such as ableism, social isolation, lack of community support, insufficient workplace accommodations, or barriers to healthcare access. While we must examine these underlying systemic issues and reduce the conditions that make disabled users dependent on potentially unreliable technologies, we must also recognize that GenAI will continue to be important for disabled users and invest in making these systems more trustworthy.

#### Trust by Necessity:

Unlike voluntary technology adopters who can abandon systems they do not trust, many disabled users face a dilemma where GenAI tools represent their best available option for accessing information, communication, or task completion. We saw that these necessities come from gaps in legal accommodations and systemic failures to provide adequate accessibility support, forcing disabled people to rely on GenAI as a substitute solution for barriers that should not exist in the first place. The specific tradeoffs users faced differed across disability identity and use cases. For instance, some participants used GenAI for support in neurotypical communication styles, despite concerns about authenticity. BLV users may depend on GenAI for object recognition while also having concerns about data privacy. These variations suggest a need for risk-aware design that can be personalized to specific users.

Sometimes GenAI is used in risky situations because the alternative is even riskier. For example, advice to consult a doctor may be impossible for users facing healthcare barriers, long wait times, or providers unfamiliar with their specific conditions. Rather than simply refusing to answer certain queries, or answering all queries equally, deep user research is needed to develop GenAI supports that meet users where they are and provide the best possible result. For example, disability-aware GenAI healthcare tools might include text simplification services, provide warnings about the relevance of results to individual situations and interactions with other medical conditions, and leverage traditional fact-checking methods to help verify that AI outputs are not hallucinations. It could also highlight opportunities for additional support and connect users to multiple pathways for professional consultation, including telehealth options, disability-specialized providers, and pharmacy consultation services.

#### The Uneven Cost of Quality:

Participants reported the need to fact- and quality-check GenAI responses or not use GenAI in high-stakes situations due to inaccurate outputs, which created extra labor and cognitive load. As GenAI is increasingly integrated into everyday settings, such as Google search results, there is also a risk that users for whom verification is more difficult are more exposed to the risk of trusting incorrect information. For example, users who need outside help from other people, or with executive dysfunction, may face higher barriers because of the meta-cognitive demands of assessing GenAI reliability, while those with memory impairments or brain fog encounter a barrier with tracking outputs they have already verified. This conditional misrepresentation deepens these barriers, as users must not only assess source credibility but also evaluate contextual appropriateness. While previous literature suggests that GenAI should route back to its information sources (e.g., website, citations) [46, 71], this still requires extra steps to verify the specific information and its relevance to the context of the current query. A more accessible interface should include clear visual and textual representations of source data, embedded within the interface. This could include side-by-side source comparison tools, automated cross-referencing across multiple sources, or even integration with disability-specific databases and resources.

Future research should examine how user-developed strategies scale with evolving GenAI capabilities, how they might be transferred or taught to other users, and what organizational or community support might enhance individual verification strategies. Studies should also examine how verification and privacy strategies vary across disability communities and GenAI tasks, and how we can move beyond forcing disabled users to choose between unreliable GenAI assistance and no assistance at all, toward a space where GenAI represents one reliable option within a robust ecosystem of accessible support and services.

### 5.2 Accessibility in and through Identity Management

While accessibility and accessibility technology are often defined in terms of how technology extends functional capabilities, this is a narrow definition that recent work has broadened significantly. Mack et al. introduced the role of accessibility tools in mitigating future impacts, reducing symptoms, and reducing accessibility taxes associated with bad usability [53]. Hsueh et al. discuss access as folk bridgecraft for overcoming accessibility gaps, as well as how selective compliance, strategic refusal, and covert disobedience impact accessibility practices in the face of emotional or other harms and loss of agency in access contexts [42].

In our findings, access is conditional, negotiated, and shaped by identity. For some, this meant having their unique linguistic practices carried through inputs and outputs, as well as having disability identities and gender, race, or linguistic practices explicitly acknowledged, affirmed, and respected in interactions. At other times, GenAI tools reinforced representational inequities (e.g., defaulting to monolithic White, English-speaking, cis-male portrayals) [39] or relied on reductive stereotypes that flattened the complexity of disabled lives. These instances raise a critical question: *Is such conditional or partial support truly access?* When access is only achieved at the expense of other equally salient identity dimensions, we must ask if it can be *fully* understood as access in any just sense. We surface insights for both designers and researchers, identifying new GenAI design and research areas centering intersectionality.

#### Access Should Be Defined by the User:

In our results, people’s conceptualizations of what it means for an interaction to be defined as accessible varied from person to person. Accessibility is achieved both through preserving one’s intersectional identities as well as leaving them out. For example, one participant (see Section 4.3.2) described these very erasures as beneficial, interpreting GenAI’s “neutralizing” of their non-normative communication styles as a way to strategically blend in or create a feeling of linguistic acceptance. However, for others this “neutrality” reflects and re-inscribes dominant linguistic, racial, and gendered norms and hierarchies seen in broader societal practices, positioning them as defaults under the disguise of neutrality. The conditional and dynamic ways disabled participants articulated both benefits and challenges of how their identities were preserved, validated, or overlooked point to a critical design implication. GenAI systems must mirror this dynamic nature by supporting *personalization features* that are tailored and responsive to users’ preferences for how they wish to be represented and responded to during their interactions. Essential information, such as information about user access needs, and their gender, race, or language preferences, should be easy to add as context that will be maintained over time. Designing for such variability shifts away from one-size-fits-all defaults and instead acknowledges that access is situated and, at times, identity-dependent.

#### Access Should Redistribute the Labor of Identity Navigation:

Prior work has examined code-switching and masking in technology interactions for neurodivergent communities [30] and older adults [40], primarily focusing on linguistic adaptations to improve system understanding [40, 61]. We similarly found GenAI-mediated code-switching and masking were used to make writing more “professional” in different contexts. However, our findings demonstrated GenAI supported additional benefits of code-switching such as interpersonal safety—a common motivation for code-switching and masking in broader social contexts [59, 72]. As such, GenAI functioned as a mediating buffer against both social and psychological harms, enabling users to engage in interactions that would otherwise be of risk. In one notable participant account, GenAI initiated these practices. While not indicated by them, the automatically standardized outputs aligned with their goals, offering a sense of linguistic acceptance and reducing the stigma of being penalized for difference. Research should examine how diverse disabled people engage in code-switching practices across different socio-technical contexts, the perceived benefits and tensions that accompany these practices, and appropriate defaults and settings for code-switching support in the context of external communication support.

#### Access Should Not Require Code-Switching:

Further, the ability to be themselves without code-switching was important to users even when confined to GenAI communication. In that context, it reduced the labor of identity navigation, led to a sense of companionship, and reduced feelings of dependence and judgment—feelings often associated with asking others for help [20]. GenAI, even with its biases, offers a low-stakes space for personal expression due to the absence of long-term social consequences and of interpersonal scrutiny (which can also amplify self-stigma [35, 41]). This led to a sense of relief for participants who otherwise needed to constantly take on labor to adjust their communication. Again here, participants described instances of automatic support in highly positive terms.

### 5.3 Reducing the Accessibility Tax

Our findings highlight the wide variety of accessibility taxes that participants pay when using GenAI, including financial costs, disclosure costs, and time spent on prompt engineering, priming and training, and verification. Here, we highlight opportunities to reduce some of these costs.

#### Reduce Excess Labor by Going Beyond WCAG Compliance:

Current accessible GenAI discussions focus on whether core tasks, such as receiving query results and viewing the query history, are accessible [60]. However, our data demonstrate the importance of access to privacy controls and information about data use and training policies, so that users can make informed decisions about GenAI use and reduce the possibility of harm. Participants also told us that when GenAI modified outputs based on their specific disability and other identity needs, GenAI could provide more accessible outputs, such as not answering a question from a blind person about which items are selected using color (*“The items with the red check marks”*) or answering a question more effectively for *“a Black person with a disability”* or modifying language and style to meet linguistic needs. GenAI should generate results that match user access needs (such as changing modality or simplifying text), and prioritizing availability of and control over GenAI settings.

#### Reduce Excess Labor Associated with Intersectional Identities:

GenAI perpetuated existing labor inequities that scholars have identified in broader social contexts, such as the “Black tax” [66, 81], through tools perpetuating unrepresentative, biased, and stereotyped outputs alongside erasing associated ethnolects. Participants described extra work to achieve outputs that captured their intersectional identities; even then, outputs often flattened their nuanced and dynamic experiences. A particularly salient example arose through multilingual and multidialectal accessibility taxes. Multilingual participants using high-resourced languages (e.g., Spanish) described systems that could preserve their linguistic identities without additional effort—the language carried through naturally into outputs. By contrast, multidialectal participants (e.g., those using African American Vernacular English and Pidgin English) reported linguistic variations either were omitted in the output unless explicitly specified or only carried through when deliberately requested. This forces low-resourced multilingual and multidialectal disabled people to invest extra labor to sustain their identities in outputs if desired. This asymmetry highlights how GenAI systems automatically sustain certain high-resourced linguistic practices while neglecting others, thereby reinforcing broader societal language hierarchies [73] and adding to the costs of access for specific subgroups. Together, these findings complicate the notion of accessibility tax by showing how GenAI systems reproduce and intensify existing inequities for multiply marginalized disabled people, posing unique accessibility taxes on them that are directly tied to their intersecting identities. Future research must interrogate how GenAI tools can reduce accessibility taxes faced by disabled people who have intersecting marginalized identities, such as racial, linguistic, gender, and queer identities.

## 6 Limitation

Our study presents several limitations, offering key opportunities for future work. First, our study is scoped to Western and US-centric perspectives. Conceptualizations of disability vary globally [10], shaping experiences of disability, accessibility, and other identity constructs. We expect these differences shape patterns of GenAI adoption, resistance, and use. Future work should seek to understand global GenAI practices among disabled people. Second, our work aims to understand GenAI use across disabled people. However, recruitment constraints led to some disability, racial, gender, and language groups being more represented than others. Moreover, our analysis does not fully account for other intersecting axes of identity, such as religion, class, and identities that fall outside of conventional demographic categories (e.g., bodytype identity). Future work should examine how these dimensions intersect with disability to shape GenAI use and resistance. Third, only three participants actively resisted GenAI use, limiting our understanding of this perspective. Yet, we understand that resistance is not merely non-use—it often reflects deeply situated concerns. Future research should further examine potential harms, reasons for resistance or desistance of use, the contexts where these are sustained, and how intersecting identity dimensions inform such decisions. For example, participants described how GenAI replaced human relationship management and its costs, including feeling like a burden to others and navigating stigmatizing social ramifications of dependence [20, 35]. However, this removes opportunities for shared learning, negotiation, and interdependent care [7].

## 7 Conclusion

GenAI holds great potential for enhancing accessibility for disabled people, while also posing new and compounded risks. Through seven cross-disability focus groups (N=20), our study highlights the role of GenAI in everyday accessibility usage among diverse disabled people—offering meaningful support and amplifying risks. We first reveal existing and new ways GenAI supports autonomy, efficiency, and communication while also introducing compounded accessibility taxes and ethical dilemmas. Second, we reveal how trust in GenAI was conditional—often shaped by necessity, negotiated through ongoing verification work, and constrained by concerns over reliability, ethical safeguards, and opaque data practices. Finally, we identify identity-based benefits and harms, where some participants articulated GenAI preserving and validating their intersecting identities, while for others, tools misrepresented, distorted, and erased them.

We provide design implications for creating transparent and trustworthy GenAI tools for multiply marginalized disabled people and encourage further research on how “access” is conceptualized and negotiated in GenAI interaction. Our findings also point to larger systemic oversights, including the need to fund disability-focused GenAI research, require accessibility considerations in development, and ensure input from disabled users in regulatory processes.

## Figures and Tables

**Table 1:** GAI use by disabled people.

	Focus	LLM	T2I	I2T	Surv	Inter/Oth	Domain	Identity Analysis
Atcheson [5]		X	X		16	21	University Students	
Pierres [68]		X				33	University Students	
Bennett [9]		X	X			10	Creatives	
Mullen [65]		X				18		
Carik [13]	ND	X	X			55,114 posts		
Glazko [30]	ND	X				8 authors	Tech experts	Race & Language
Xie [95]	BVI			X		14		
Adnin [1]	BVI	X		X		19		Race & Gender
Tang [86]	BVI	X	X	X		20		
Huffman [43]	DHH	X			80	9		Language & Ethnicity

LLM=Large Language Model. T2I = Text to Image and I2T = Image to Text conversion. Sur(vey), Inter(view)/Oth(er) list number of participants. ND = Neurodivergent; BVI = Blind & visually impaired; DHH = d/Deaf or hard of hearing. Identity analysis studies discussed how these identities impacted GAI use in results.

**Table 2:** Participant Demographics. All pseudonyms were made up by the participants or author.

Name	Gen.	R/E	Language	Disability (self-reported)	GenAI Use
Alexander	M	Black	English	I have mild dyslexia	ChatGPT, Gemini, MetaAI
Lex	NB trans	Hispanic; Indigenous	Engish	Wheelchair user, chronically ill, SMI, neurodivergent, osteoarthritis in all my bones	ChatGPT
Emory	NB trans	White	English	Long COVID, ADHD, PTSD, Speech Impediment	ChatGPT
Fran	NB	White	English	I have a mix of gastrointestinal and psychological disabilities	ChatGPT, Claude, Grammarly, Notion
Tessa	F	White	English	Low vision	BeMyAI, SeeingAI, Envisions AI, Eventbrite, ChatGPT
Bond	M	Asian	English, Gujarati, Hindi	Congenital central hyperventilation syndrome	GenAI Non-use
Sara	F	White	English	I am a paraplegic since birth with several spinal defects, including scoliosis	Claud, ChatGPT, Perplexity, MetaAI, Descript
Tonya	F	Black; Indigenous	English	I have ADHD and also diabetes	ChatGPT, MetaAI, Midjourney
Amy	F	Black	English	I am deaf and use sign language, assistive technology and lip reading to communicate	ChatGPT, Gemini
Kim	F	Indigenous	English; Spanish	I experience chronic fatigue, which affects my energy levels and ability to perform daily tasks. Dyslexic	ChatGPT (voice), Gemini
Michelle	F	Black	English	noise sensitivity	CanvaAI, Adobe Illustrator, Photoshop, Claude, ChatGPT
Ann	F	Black	English, AAE	Chronic pain	Grammarly, ChatGPT, CanvaAI, DALL-E, Claude, Gemini
Sophia	F	Asian	English	Severe hearing loss	GenAI Non-use
Jasmine	F	Black, Hispanic	ASL, SEE, English, Spanish	Deaf, GAD, PTSD, FMS, ADHD	GenAI Non-use
Nicole	F	White	English	Totally blind with a significant hearing loss	ChatGPT
Emma	F	Asian	English, Hindi, Urdu	I am blind	BeMyAI
Thomas	M	Hispanic	English, Spanish	I have Ushers Syndrome type 2 which has led to the loss of my vision and partial loss of my hearing.	SeeingAI, BeMyAI, ChatGPT
Morgan	F	Spanish, South Am. Indian	English	I am blind	ElevenLabs, SeeingAI, BeMyAI, PicCybot, ChatGPT
Ethan	M	Hispanic, Indigenous, White	English, Spanish, Japanese	DeafBlind^6^, Polyneuropathy mobility, dexterity, tactile and balance deficits, speech disability	AlanAI, BeMyAI, PrivateGPT, Llamafile
Maddy	F	White	English	Learning disabilities and autism; AAC user	GoogleAI overview, ChatGPT

Gen(der): NB= non binary. R(ace)/E(thnicity): Hispanic = Hispanic or Latino or Spanish; Indigenous = Indigenous American or Alaskan Native; Language: AAE=African American English, SEE = Signed Exact English; Disability: GAD = General Anxiety Disorder, FMS = Fibromyalgia Syndrom; SMI=Serious Mental Illness

**Table 3:** Overview of participants’ occupational, educational, and technical backgrounds. Expertise were defined as follows: Novices: I need a lot of help; Beginner: I can do some things if I get help; Intermediate: I can do many things on my own; and Expert: I can do hard things and help others too. The first four items came from the optional post-study survey (N=10/20); the remainder from the main study (N=20/20). Use types were categorized use into four primary areas: creative outlet, personal, professional and academic support, information seeking, and accessibility assistance.

Topic	Responses
GenAI Experience	Novices (3); Beginner (1); Intermediate (4); Expert (2)
Profession (N=10/20)	Education (1); Healthcare or social services (2); Technology (e.g., software, IT, digital tools) (2); Arts, media, or design (1); Retail, food service, or hospitality (1); Government, public service, or nonprofit (1); Science, research, or engineering (2); Business, finance, or administration (1); Manufacturing, construction, or skilled trades (1); I have not worked in a paid job (1)
Employment Status (N=10/20)	Employed full-time (4); Employed part-time (1); Self-employed or freelance (1); Not currently working (unemployed and looking for work) (1); Student (2); Retired (1)
Education Level (N=10/20)	High school diploma or equivalent (e.g., GED) (1); Some college, no degree (1); Bachelor’s degree (BA, BS) (4); Graduate or professional degree (e.g., MA, MS, MBA, JD, PhD, MD) (4)
Use Types (N=20/20)	Creative (e.g., image generation, creative writing, generate voices) (11); Professional & Academic support (e.g., cover letters, programming, brainstorming) (6); Information Seeking (e.g., search, therapy questions) (2); Personal (e.g., emotional conversations, tax support); (5) Accessibility Assistance (e.g., compose messages, job applications, navigating spaces, healthcare questions, coding/programming, reading documents, image descriptions, accessibility remediation) (13)

## CCS Concepts

• Human-centered computing → Empirical studies in accessibility.

## Footnotes

1 In this paper, we used participant-preferred language when shared, otherwise we defaulted to disability-first language. The choice between disability first and people-first language remains an ongoing discussion within the HCI/ ASSETS community [76].

2 Code-switching refers to linguistic, behavioral, and presentation adaptations people make to align with dominant norms and navigate social power dynamics in the context of their environment [58].

3 Masking refers to the suppression or alteration of neurodivergent traits to conform to social expectations, avoid judgment, or be perceived as neurotypical [15, 70].

4 We defined AI as: Technology that helps computers and machines act more like a human brain. They can solve problems, make decisions, learn new things, and understand information.

5 We defined GenAI as: A type of AI technology that creates new content (e.g., text, images, video, music, etc.) based on the information the user provides.

6 Category 3 Blindness Both Eyes and Auditory Processing Disorder with Decoding Deficit

7 An ongoing process of deciding how to move toward access first defined in [52].

8 “Crip” having been in use since at least 1979 [51].

9 A search of the ACM digital library found only one mention, in the context of DIY accessibility solutions in the workplace [14].

## A Appendix: Focus Group Questions

### Section 1: GenAI for Access

- What GenAI tools do you currently use in your day-to-day life?
- What GenAI tools do you use for accessibility purposes?
- Tell us about a time you used GenAI and it was beneficial.
- Tell us about a time a GenAI tool was challenging to use or not accessible.
- Based on your current understanding and/or experience of GenAI, what do you think could be the potential benefit of GenAI?

### Section 2: Trust and Safety

- Tell us about a time when you felt you could really trust a GenAI tool?
- Tell us about a time when you felt you could not really trust a GenAI tool?
- Do you have any concerns about sharing personal information about your disability with GenAI tools?
- How do you determine whether AI-generated content about disability related topics are accurate?
- Are there any features or tools that would make you more secure in the information AI generates?

### Section 3: Identity and GenAI Use

- Tell us about how your identity facets, such as your race, language, and gender or sexuality, shape the GenAI tools you adopt and how you use these GenAI tools for accessibility?
- Tell us about times where you felt the GenAI tool or specific usage honored your identities?
- Tell us about times where you felt the GenAI tool or specific usage felt in conflict with your identities?
